# Women Have More Recurrences of Atrial Fibrillation than Men after Thoracoscopic Ablation and Suffer More from Established Risk Factors

**DOI:** 10.3390/jcm12072650

**Published:** 2023-04-02

**Authors:** Robin Wesselink, Bente Mossink, Eva R. Meulendijks, Nicoline W. E. van den Berg, Jolien Neefs, Makiri Kawasaki, Benedetta Fabrizi, Femke R. Piersma, Rushd F. M. Al-Shama, Tim A. C. de Vries, Jonas S. S. G. de Jong, Wim Jan P. van Boven, Antoine H. G. Driessen, Joris R. de Groot

**Affiliations:** 1 Department of Clinical and Experimental Cardiology and Cardiothoracic Surgery, Heart Center, Amsterdam UMC, University of Amsterdam, Meibergdreef 9, 1105 AZ Amsterdam, The Netherlands; 2 Amsterdam Cardiovascular Sciences, Heart Failure and Arrhythmias, Meibergdreef 9, 1105 AZ Amsterdam, The Netherlands; 3 Department of Cardiology, Rijnstate Hospital, Wagnerlaan 55, 6815 AD Arnhem, The Netherlands; 4 Department of Cardiology, Onze Lieve Vrouwe Gasthuis, Oosterpark 9, 1091 AC Amsterdam, The Netherlands

**Keywords:** sex differences, atrial fibrillation, thoracoscopic AF ablation, risk factors

## Abstract

Introduction. Atrial fibrillation (AF) is more prevalent in men than in women. However, women with AF are more symptomatic, have a worse quality of life, a higher stroke risk and may therefore benefit most from ablation. In this study we aim to identify the risk of recurrent AF after thoracoscopic ablation, and assess the differential impact of the risk factors for recurrence between women and men. Method. This is a single center cohort study, including patients undergoing thoracoscopic ablation for advanced AF between 2008 and 2019. All patients were clinically followed up for two years with quarterly 24 h Holter monitoring and ECGs for the detection of recurrent AF. Left atrial appendage (LAA) tissue was collected for collagen analysis. Results. We included 571 patients, of whom 143 (25%) were women. Women were older than men (63 ± 8.3 y vs. 59 ± 8.5, *p* < 0.001), but had fewer cardiovascular risk factors, myocardial infarctions (1.4% vs. 6.5%, *p* = 0.03) and, in particular, vascular disease (7.0% vs. 16.1%, *p* = 0.01). Women suffered more from AF recurrence, driven by more atrial tachycardias, and sex was an independent risk factor for recurrence (HR1.41 [1.04–1.91], *p* = 0.028]). The presence of vascular disease was associated with an increased risk for AF recurrence in women, but not in men. In LAA histology, women had more collagen than men, as had patients with persistent compared to paroxysmal AF. Conclusion. Women had 15% more recurrences, driven by more atrial tachycardias, which may be explained by a more fibrotic atrial substrate. What’s new? Women undergoing thoracoscopic AF ablation have a higher risk of recurrent AF, driven by more atrial tachycardias. Among patients with left atrial enlargement or persistent AF, women have worse outcomes than men. Vascular disease was a risk factor for recurrence in women, but not in men. In a histopathologic analysis of the left atrial appendage, women had more collagen than men, as had patients with persistent compared to paroxysmal AF.

## 1. Introduction

Atrial fibrillation (AF) is the most common cardiac arrhythmia with a prevalence of 2–4%, which is expected to increase due to the ageing of the general population [1,2]. AF affects men more often than women (596 per 100,000 vs. 373 per 100,000) [3]. However, as women tend to outlive men, the lifetime risk of AF (>30%) is the same for women and men [4]. Additionally, rhythm control treatment (medication or ablation) of AF is less effective in women compared to men [5], suggesting that the mechanisms of AF or AF recurrence in women may differ from those in men.

Sex-specific mechanisms of AF have only been sparsely studied. In women, multi-parity has been associated with an increased risk of AF [6]. Sex hormones seem to be involved in the genesis of AF, due to the increase of AF incidence after menopause [7]. However, the effect of sex hormones and hormone replacement therapy on AF remains unclear due to conflicting reports [8,9]. Meanwhile, women with AF suffer from a relatively greater symptom burden and lower quality of life [10,11]. Secondly, women have reduced efficacy in AF treatment [12,13], and an increased stroke risk [7,11,14,15]. Women tend to suffer from AF at an older age than men [10], at which time their clinical characteristics may be less favorable for successful treatment. Women treated with catheter ablation have a higher risk of complications, such as cardiac tamponade and bleeding, and a higher risk of hospitalization for AF [15,16]. Finally, women are less likely to receive rhythm control therapy than rate control therapy [10,17]. Moreover, women undergo fewer repeat ablations, which may suggest that women are more likely to be switched to a rate control strategy, as they do not have fewer AF recurrences after ablation [18].

In summary, women seem to respond less to rhythm control treatment, possibly due to our incomplete understanding of the mechanism underlying AF in women versus men. This incomplete understanding may be the result of the underrepresentation of women in clinical trials [15]. In standard clinical care, presumptions about treatment efficacy, risk factors for AF or AF recurrence are often generalized in women, but may not be fully valid.

Here, we studied the differences in clinical characteristics, and the specific impact of the established risk factors for AF recurrence between women and men undergoing thoracoscopic ablation, making use of a well-characterized cohort of patients with advanced AF [19] with standardized two years follow-up for arrhythmia recurrence.

## 2. Methods

We included patients undergoing thoracoscopic AF ablation at the Amsterdam UMC location Meibergdreef, in the Netherlands. Patients were treated between November 2008 and March 2019, and were included in the AFACT trial [19], the MARK-AF registry study (NL50069.018.14) or undergoing thoracoscopic AF ablation as standard treatment (n = 107). The institutional review board waived the need for informed consent for the analysis of clinical data in the latter group of patients. As part of the preoperative work-up, all patients underwent a transthoracic echocardiogram (TTE).

Patients selected for thoracoscopic AF ablation usually have persistent AF, an enlarged left atrium, a previously failed catheter ablation, or patient preference of thoracoscopic ablation instead of a catheter ablation [19]. All patients underwent thoracoscopic AF ablation, with bilateral pulmonary vein isolation (PVI) and left atrial appendage (LAA) exclusion, as previously described [20]. In patients with persistent AF, defined as continuous AF for at least seven days, according to the ESC guidelines [15], an additional roofline (connecting both pulmonary vein lesions) and trigone line (connecting the roofline to the left fibrous trigone at the aortic annulus) were constructed (Dallas lesion set) [21]. Conduction block of all lesions was confirmed during the surgical procedure, as previously described [22]. Patients participating in the AFACT trial were randomized for additional ganglion plexus (GP) ablation versus no additional GP ablation [19]. As the AFACT trial did not show a difference in AF recurrence between treatment arms, the data of patients with and without GP ablation were pooled. In all patients, the LAA was excised and retrieved, and a subset histopathological analysis was performed. Postoperatively, patients were not admitted to the ICU, but only to the postoperative recovery room. Generally, patients were discharged for home care on the third day after the procedure.

## 3. Clinical Follow-Up

Three months after the procedure, all antiarrhythmic drugs (AADs) were discontinued. If patients were in AF, they underwent electrical cardioversion (ECV). Patients were followed up at the outpatient clinic every three months for two years, with electrocardiogram (ECG) and 24 h Holter monitoring for the detection of recurrent AF. Anticoagulation was discontinued only in patients with CHA2DS2-VASc scores of 0 (or 1 in women) at 6 months follow-up. Recurrence of AF during two years follow-up was defined according to the HRS/EHRA/ECAS consensus statement as any atrial tachyarrhythmia documented on ECG or lasting longer than 30 s on continuous monitoring without AAD therapy [23]. Recurrences during the blanking period, the first three months after the procedure, were not considered a failure of the procedure. All outcomes were adjudicated by a blinded and independent electrophysiologist, unaware of the patient’s sex or clinical characteristics.

## 4. Histological Analysis of Atrial Interstitial Collagen

Part of the excised LAA was fixed in 4% formalin and embedded in paraffin. Sections with a 5 µm thickness were prepared and stained with Picrosirius red. The percentage of interstitial collagen (red) and myocardium (yellow) were quantified. Sections were digitized at 40× magnification (Philips IntelliSite Ultra Fast Scanner, 0.25 µm/pixel) and 20 non-overlapping fields (maximal 5000 by 5000 pixels) from each patient were randomly selected for collagen quantification. Endocardial, epicardial and perivascular collagen were manually selected. An automated image analysis using Image J (version 1.50i) color deconvolution was performed to determine the area fraction of collagen of the combined area of cardiomyocytes and collagen at an automatic and at a maximized threshold value.

## 5. Statistical Analysis

Data were presented as the mean ± standard deviation (SD) for the normally distributed values, and median [interquartile range] for not normally distributed values. Binomial or categorical values were presented as a number (percentage). Because only a few patients had longstanding persistent AF, we categorized those patients as persistent AF. Associations of the baseline characteristics with recurrence of AF were performed with a univariate Cox proportional hazards model. Variables with *p* < 0.1 and no missing data were included in the multivariable Cox proportional hazards model. The cross correlation of the variables used in the Cox proportional hazard model was assessed with a logistic regression for binomial variables and a linear regression for continuous variables. Association of binary variables with a recurrence of AF was performed using the Pearson Chi-squared test or Fisher’s exact test if the expected values were under 5. Pairwise exclusion was used in the case of missing data. All performed tests were two-sided and a *p*-value of <0.05 was considered statistically significant. All analyses were performed with R (RStudio Team (2020), version 1.3.1093).

## 6. Results

We included 571 patients, 207 with paroxysmal AF and 364 with persistent AF, of whom 143 (25%) were women (52 (36.4%) with paroxysmal AF and 91 (63.6%) with persistent AF, who underwent thoracoscopic surgical AF ablation. There were 108 (18.9%) patients who had undergone a previous catheter ablation for AF, of whom 56 (52%) had undergone one procedure, 36 (33%) had undergone two, 10 (9%) had undergone three, 2 (2%) had undergone four and 4 (4%) had undergone five previous catheter ablations. Women and men had equal rates of previously failed catheter ablation (18.5% vs. 20.3%, *p* = 0.72). Both women and men had AF for 4 [2, 8] years. At the time of the procedure, women were older than men (63 ± 8.3 year vs. 59 ± 8.5, *p* < 0.001), but had fewer cardiovascular risk factors; fewer myocardial infarctions (1.4% vs. 6.5% *p* = 0.03) and less vascular disease (history of myocardial infarction, percutaneous coronary intervention or peripheral artery disease) (7.0% vs. 16.1% *p* = 0.01). Women more frequently had moderate to severe mitral valve insufficiency (44 (31.0%) vs. 83 (19.6%), *p* = 0.007). There were no differences in the prevalence of hypertension, history of heart failure or diabetes.

Women had a shorter stature (1.70 ± 0.07 vs. 1.84 ± 0.07 m, *p* < 0.001) and a lower body weight (79.0 ± 14 vs. 93.7 ± 13.3 kg, *p* < 0.001), but on average both women and men were overweight (BMI 27.3 ± 4.5 vs. 27.7 ± 3.6 kg/m^2^, *p* = 0.24). Left atrial volume index (LAVI, 42.6 ± 13.5 vs. 42.0 ± 12.3 mL/m^2^, *p* = 0.65) and left ventricular ejection fraction (LVEF, 53 ± 10 vs. 52 ± 10, p = 0.36) were the same in women and men, respectively. Women had higher serum CRP (2.0 [0.9–4.28] vs. 1.3 [0.65–2.9] mg/L, *p* = 0.006), more often used loop diuretics (22 (15.4) vs. 36 (8.4), *p* = 0.026), and had higher serum N-terminal pro-brain natriuretic peptide levels (NT-proBNP) (425 [180–916] vs. 256 [101–618], *p* < 0.001). These and other baseline characteristics are shown in Table 1.

## 7. Freedom of AF Recurrence

**Figure 1:**
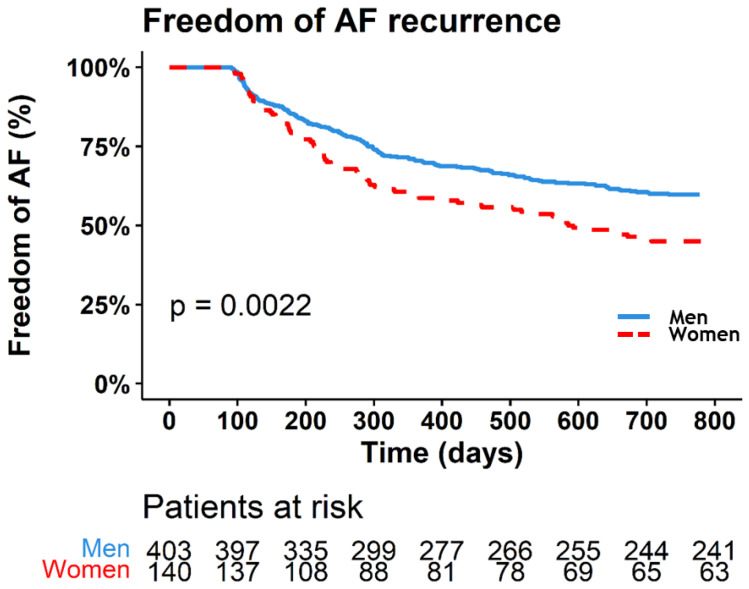
Kaplan–Meier analysis of women and men during two years follow-up. Men: solid blue line, women: dashed red line. AF = atrial fibrillation.

Following thoracoscopic AF ablation, 94.9% of patients completed two year follow-up, 28 subjects (4.9%) were lost to follow-up. The overall rate of freedom of AF after two years was 56%, which was lower in women than in men: 45% of women were free from AF recurrence compared to 60% of men (*p* = 0.002) (Figure 1). The difference in AF recurrence was mainly driven by a higher proportion of atrial tachycardias (ATs) in women (53 (37.9%) vs. 86 (21.2%), *p* < 0.001), whereas the proportion of AF as a first recurrence was the same (26 (18.6%) versus 76 (18.9%), *p* = 1) (Table 2).

## 8. Association of Variables with Recurrence of AF

**Figure 2:**
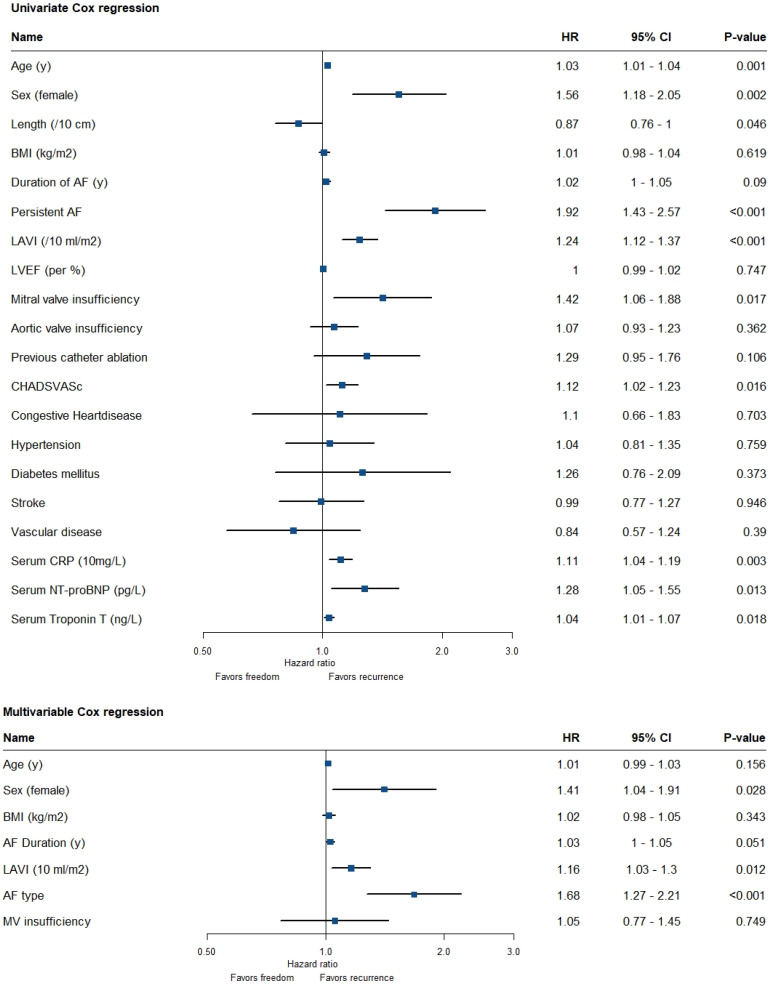
Univariate and multivariable Cox regression of patient characteristics and freedom of AF recurrence, for all included patients. AF atrial fibrillation, BMI body mass index, CI confidence interval, cm centimeter, CRP c-reactive protein, HR hazard ratio, kg kilogram, LAVI left atrial volume index, LVEF left ventricular ejection fraction, m meter, mL milliliter, MV mitral valve, ng nanogram, NT-proBNP n-terminal pro-brain natriuretic peptide, pg pictogram, y years, µg microgram.

**Figure 3:**
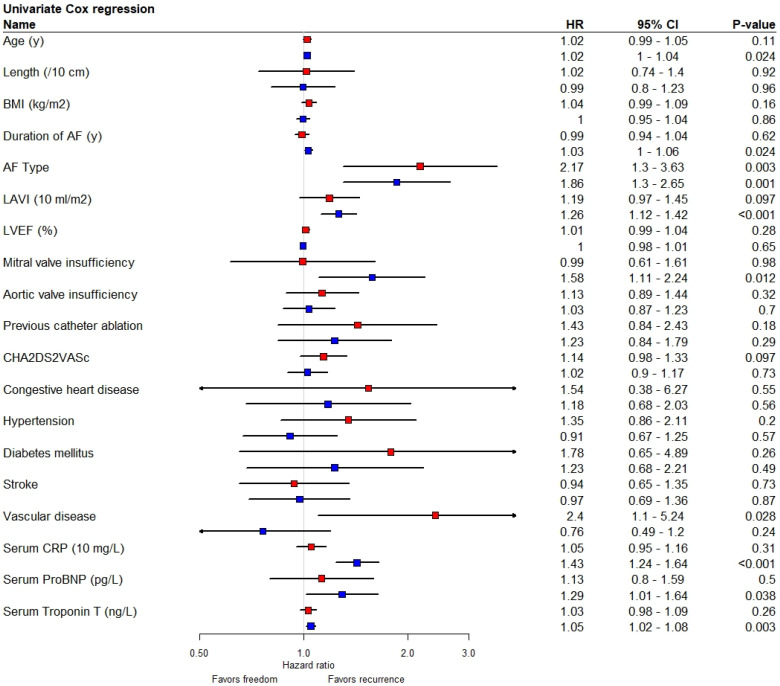
Univariate Cox regression of patient characteristics and freedom from AF recurrence for women (first, red) and men (second, blue) separately. AF atrial fibrillation, BMI body mass index, CI confidence interval, cm centimeter, CRP c-reactive protein, HR hazard ratio, kg kilogram, LAVI left atrial volume index, LVEF left ventricular ejection fraction, m meter, ml milliliter, MV mitral valve, ng nanogram, NT-proBNP n-terminal pro-brain natriuretic peptide, pg pictogram, y years, µg microgram.

**Figure 4:**
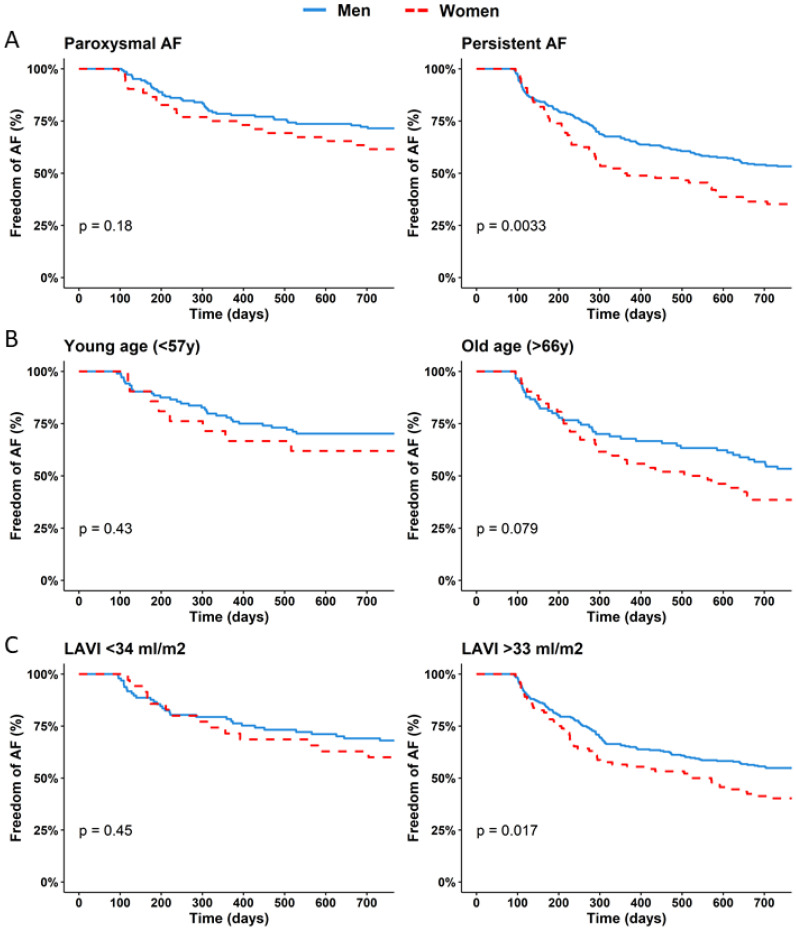
Different impact of the risk factors between women and men. (**A**) Patients with paroxysmal AF (left) and persistent AF (right); (**B**) young (first quartile, <55 y) and old patients (fourth quartile, >67 y); (**C**) normal LA size (LAVI < 34 mL/m^2^) and enlarged LA (LAVI > 33 mL/m^2^).

Women were at higher risk for recurrent AF (HR 1.56 [1.18–2.05], *p* = 0.002) (Figure 2). Sex was independently associated with AF recurrence (HR 1.41 [1.04–1.91], *p* = 0.028), corrected for age, BMI, AF duration, LAVI, AF type and mitral valve insufficiency. Figure 3 shows a forest plot with separate rows for women and men, demonstrating the differential effect of the risk factors between sexes. The risk factors, including age, persistent AF and LAVI appear to affect women and men in the same direction and with the same magnitude. Both women and men with persistent AF had an increased risk of recurrent AF. The additional risk of recurrence in patients with persistent AF compared to paroxysmal AF was 92% (HR 1.92 [1.43–2.57], *p* < 0.001). This translates to 26% more recurrences in women (38.5% vs. 64.8%) compared to 18% in men (28.5 vs. 46.7) (Figure 3). The Kaplan–Meier analysis in Figure 4A shows that women with persistent AF have significantly more recurrences than men (64.8% vs. 46.7%, *p* = 0.003). In the same way, the Kaplan–Meier analysis in Figure 4C shows that women with enlarged left atria have more recurrences than men (59.8% vs. 45.1%, *p* = 0.012).

The assessment of the effect of the risk factors within the same sex revealed that some risk factors for recurrence affect women and men differently. Women with a history of vascular disease (n = 10, versus n = 133 without vascular disease, Table 1) have a high risk of recurrent AF (HR 2.40 [1.1–5.24], *p* = 0.03), while vascular disease in men was not associated with the risk of recurrent AF (HR 0.76 [0.49–1.20, *p* = 0.24), resulting in a significant interaction (HR 3.32 [0.30–1.41], *p* = 0.006). Mitral valve insufficiency was a risk factor for recurrence for men (HR 1.58 [1.11–2.24], *p* = 0.012), but not for women (HR 0.99 [0.61–1.61], *p* = 0.98). Of the ten included cardiovascular risk factors (CHADSVASCc components and score, valve insufficiency, BMI), women had higher point estimates in seven of those. C-reactive protein (CRP) had a significant interaction with sex (interaction term HR 0.71 [0.61–0.85], *p* < 0.001), indicating that every increase of the CRP by 10 milligram/L increases the risk of recurrence in men 29% more than women. Of note, the baseline CRP concentration was significantly higher in women than in men (Table 1).

Finally, it must be taken into account that this analysis only demonstrates the increased risk within a selected group, shows relative risks and does not reflect the absolute risk of recurrence. The HR for increased LAVI in women was numerically and not significantly lower than that of men (1.19 [0.97–1.45] vs. 1.26 [1.12–1.42]). A Kaplan–Meier analysis revealed that women and men with a normal-sized left atrium (LAVI < 34 mL/m^2^) had similar recurrence rates (40.0% vs. 32.0%, *p* = 0.45), while women with a left atrial enlargement had a higher recurrence rate than men with an LA enlargement (59.8% vs. 45.1%, *p* = 0.017). Similarly, advanced age is a stronger risk factor for women than for men: women and men aged <55 (quartile 1) had the same prognosis (38.1% vs. 29.8%, *p* = 0.43), while women aged >66 had a trend toward higher recurrence rates than men (61.5% vs. 46.6%, *p* = 0.11).

## 9. Histology

**Figure 5:**
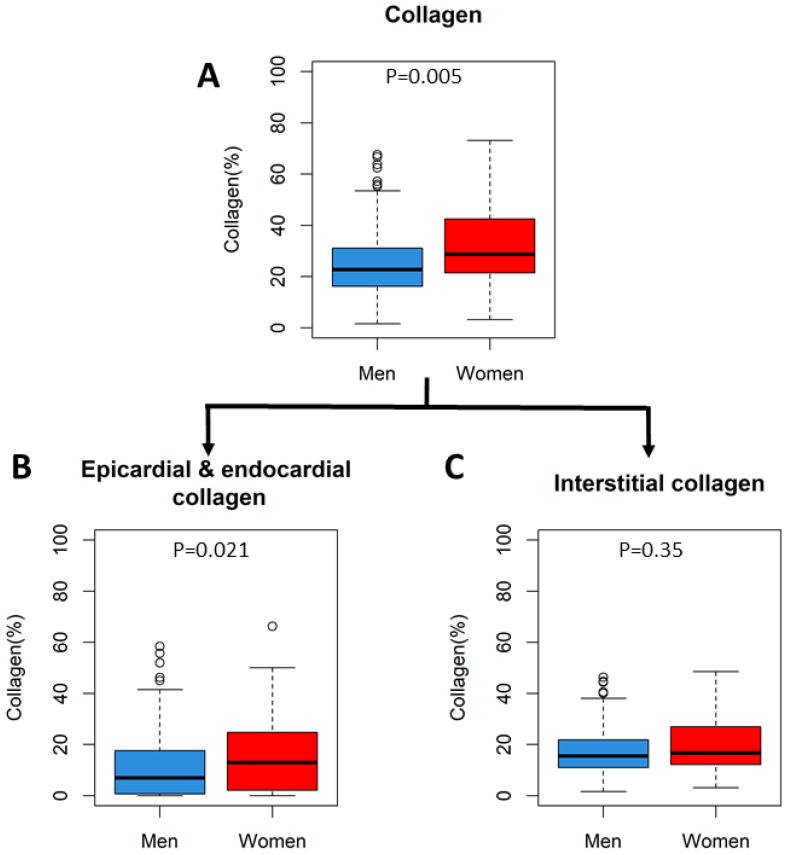
Percentage of collagen in Picrosirius red-stained histologic sections. (**A**) Total percentage of collagen in the histologic sections. (**B**) Percentage of epicardial and endocardial collagen, (**C**) Percentage of interstitial collagen.

**Figure 6:**
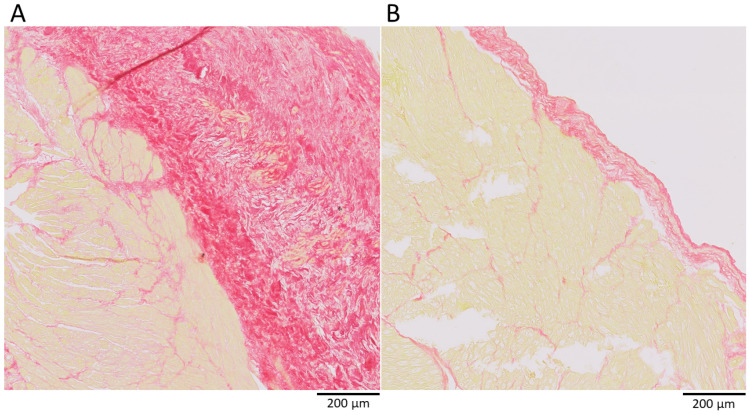
Typical example of histologic collagen analysis of the left atrial appendage tissue. Red = collagen, yellow = myocardial cells. (**A**) Relatively thick epicardial layer of a woman, (**B**) relatively thin epicardial layer of a man.

Picrosirius red stained histologic sections of the left atrial appendage were available for 209 patients. The area proportion of collagen, normalized to the area proportion of myocardium, was higher in women than in men (28.7 [21.6–42.1]% vs. 22.7 [16.2–31.1]%, *p* = 0.005). The higher proportion of collagen in women was driven by more epicardial and endocardial collagen deposition (13.0 [2.1–24.8]% vs. 6.9 [0.7–17.6]%, *p* = 0.021), since the proportion of interstitial collagen was equal between women and men (16.7 [12.2–26.9]% vs. 15.5 [11.0–21.8]%, *p* = 0.35]) (Figure 5). A typical example is shown in Figure 6. Patients with persistent AF had more collagen compared to patients with paroxysmal AF (26.7 [17.1–38.6]% vs. 21.0 [153–27.7]%, *p* = 0.002). This was driven by more interstitial fibrosis in patients with persistent AF (18.2 [12.4–27.9] vs. 12.58 [8.9–17.5], *p* < 0.001). The proportions of men and women with persistent AF were the same in these groups (*p* = 0.45). Patients with and without a previously failed catheter ablation had equal amounts of interstitial (18.2 [10.9–28.1] vs. 15.4 [11.2–22.3], *p* = 0.30) and epi- and endocardial collagen (7.67 [0.0–21.2] vs. 8.7 [1.7–18.7], *p* = 0.73) Patients with AT as first recurrence had the same amount of collagen as patients with AF as first recurrence (27.6 [17.9–34.6]% vs. 26.9 [18.4–35.5]%, *p* = 0.98). The total amount of collagen (HR 0.99 [0.88–1.13], *p* = 0.93) and epi-and endocardial collagen (HR 1.00 [0.97–1.03], *p* = 0.83) were the same for patients with and without recurrence of AF.

## 10. Discussion

We present the results of a large single center cohort of women and men undergoing thoracoscopic AF ablation for advanced AF. Women had 15% more recurrence of AF than men, which was driven by more atrial tachycardias as first recurrence. Sex was an independent risk factor for recurrence after correction for age, AF duration, LAVI, AF type and mitral valve insufficiency. Furthermore, we explored that women with additional risk factors, such as persistent AF and old age, have more recurrent AF after two years follow-up compared to men. Lastly, we demonstrated that women have more atrial fibrosis, particularly more epicardial and endocardial fibrosis compared to men.

We further analyzed the recurrence rates of patients with and without aforementioned risk factors for AF recurrence, and showed that recurrence rates for young women, and women without LA enlargement are similar to the recurrence rate of old men, or men with LA enlargement. In other words, men with a risk factor may have the same prognosis as women without that risk factor. For women, the added risk of a certain risk factor for recurrence adds to the already increased risk for the female sex. Therefore, these risk factors for recurrence impact women more than men. One explanation for this (considering that sex remained an independent risk factor after correction for age, AF duration, LAVI, AF type and mitral regurgitation) is the presence of a more diseased atrial substrate observed in the histopathological analysis of women’s samples. Patients with persistent AF had more interstitial fibrosis compared to paroxysmal AF patients, while women demonstrated more endo- and epicardial fibrosis. The proportion of endo- and epicardial fibrosis, as opposed to interstitial fibrosis, were associated with increased recurrence of AF. However, in-silico modeling of AF with increased epicardial fibrosis resulted in more complex and stable AF patterns [24] This may suggest that more fibrosis, seen in women compared to men, is a sign of a more progressed, more diseased substrate. This is an observational, hypothesis-generating analysis. We can only speculate about the underlying mechanism, due to the design of this study. One explanation may be that AF is a late manifestation of atrial disease in women compared to men. Therefore, when the arrhythmia emerges in women, it may be harder to control than in men. We speculate that in women, more atrial cardiomyopathy is needed to cause AF, and that the arrhythmia manifests itself later in the course of the disease than in men. While women had more fibrosis and more atrial tachycardias than men, fibrosis was not associated with the occurrence of atrial tachycardia. Women and men had equal amounts of redo ablations during follow-up after thoracoscopic ablation. Our analysis suggests that vascular disease may be a sex specific risk factor for women, and not for men. Of note, the number of female patients with vascular disease was low, but the difference in AF recurrence risk between men and women nevertheless was significantly higher in women.

## 11. Baseline Differences

In our analysis, women treated for AF were older than men, which has been reported by others before [3,18,25]. The time from first diagnosis to treatment was the same for women and men. Women had more moderate mitral valve insufficiency, which may contribute to increased risk of AF recurrence. Women had higher serum NT-proBNP. Ongoing AF may have contributed to this difference, however, equal distribution of AF type in women and men does not indicate a different rhythm distribution during blood draw. Additionally, NT-proBNP levels normalize or at least decrease in the majority of patients 6 months after undergoing thoracoscopic ablation, suggesting that HFpEF is a reversible symptom of AF [26]. Conversely, men had more cardiovascular risk factors, more myocardial infarctions and more vascular disease. Moreover, these and other cardiovascular risk factors are associated with both AF development and ischemic stroke [27,28].

Women and men are different biological entities with unique regulatory mechanisms. In women, pregnancy and birth require great flexibility of the body’s cardiovascular system. Multiple childbirths have been associated with increased risk of AF, suggested due to physiologic and hormonal stress on the heart [29]. On top of that, irregular menarche, or early or late onset of menarche may affect the risk of AF [30,31]. While hormonal data were not available, the expected effect of premenopausal hormonal fluctuations is low. The lowest age quartile in women ranged from 40 to 58, and none were using hormone replacement therapy. The contribution of sex-specific hormones to incident AF remains sparsely studied. [8,31] The incidence of AF in pre-menopausal women is low, and increases after 50 years of age [7], which suggests an interplay between estrogen and AF. However, the effect of estrogen and estradiol on AF is complex. Especially in postmenopausal women, who experience increased blood pressure and BMI, which are established risk factors for AF [32]. Studies focused on hormone replacement therapy in women show conflicting results. Hormone replacement therapy in women after myocardial infarction may result in reduced incidence of AF [33]. Conversely, three trials investigating the effects of hormone replacement therapy on incident AF demonstrated an increased risk of AF development [8,34,35].

Thoracoscopic ablation is an effective alternative to catheter ablation, especially in patients with persistent AF [36]. Thoracoscopic ablation is more effective, but comes at the cost of slightly more complications than catheter ablation [36,37]. Stand-alone thoracoscopic ablation can be considered in patients with a previously failed catheter ablation, or for patients who prefer a surgical approach [23]. A meta-analysis performed in a catheter ablation setting shows a 25% reduced freedom of AF for women, a more nuanced difference compared to our data [13]. Moreover, women undergoing ablation have a higher risk of complications, as described in a nationwide Dutch registry (women had a doubled risk of tamponade and vascular complications, and a threefold higher risk of bleeding) [16]. A German registry (women at double risk of bleeding and pericardial effusion) [5] and an analysis of the National Inpatient Sample Database from the United States (women at 1.5 times higher risk of at least 1 major complication, among which pericardial effusion) show similar results. A recent sub analysis of the CABANA trial per sex revealed equal complication rates between women and men undergoing catheter ablation [38]. However, this was an analysis involving only 1108 patients, of whom 413 women, which is underpowered to detect differences in, for example, tamponade, which occurred just as often (0.8%) as in the abovementioned registries. The lower treatment efficacy in women versus men, in addition to a 2–3 times higher risk of procedural complications in women [5,16], may contribute to a referral bias for rhythm control therapy [39] in which either cardiologist or patient may be reluctant to choose rhythm control therapy. There are signs that early rhythm control therapy is beneficial, which in the light of our results may be particularly valid for women [40]. With a delayed ablation, the atrial substrate may undergo even further remodeling, additional risk factors for recurrence could develop, which may mitigate the chances of successful restoration of sinus rhythm. Studies investigating early interventions should therefore focus specifically on the effect in women. In any case, sex is a modulator of both the route to treatment and the outcome afterward.

## 12. Strengths and Limitations

We performed follow-up with intermittent 24 h Holter monitoring, which is a systematic error, as monitoring was the same in men and women and in patients with and without AF recurrence, but a limitation nonetheless. Indeed AF burden may be a better predictor of prognosis compared to a dichotomous outcome [41]. However, our monitoring strategy is more stringent than recommended by the HRS/EHRA/ECAS consensus document [23]. In a subgroup of patients, serum CRP and NT-proBNP levels may be more predictive of outcome than sex, while both CRP and NT-proBNP are known to be higher in women compared to men [42]. Since serum biomarkers were not available for all patients, they were not included in the multivariable regression analysis. We performed our analysis in a well characterized population of patients with advanced AF, undergoing a consistent treatment policy and with a standardized clinical and electrocardiographic follow-up for 2 years. In this study, we included patients in the course of 10 years, and the results of three study cohorts were pooled. Due to consistent inclusion criteria, and a consistent inclusion ratio of women to men, we are confident that the pooling of these cohorts has not introduced significant heterogeneity. Patients selected for thoracoscopic AF ablation have advanced AF, often with multiple comorbidities and risk factors for recurrence, which may not represent patients undergoing standard catheter ablation. The percentage of patients lost to follow-up after two years was low (4.9%).

## 13. Conclusions

Women undergoing thoracoscopic AF ablation were older, had fewer cardiovascular risk factors, but more often suffered from mitral valve insufficiency than men. Following thoracoscopic ablation, women had 15% more recurrences, driven by more atrial tachycardias, which may be associated with a more progressed atrial substrate. Furthermore, risk factors for recurrent AF impact women more severely than men, which may be overlooked by the relative risks of regression analysis.

## Figures and Tables

**Table 1:** Baseline characteristics.

	Women	Men	*p*-Value	Missing
N	143	428		
Age (y)	63.3 (8.3)	59.3 (8.5)	<0.001	
BMI (kg/m^2^)	27.3 (4.5)	27.7 (3.6)	0.243	
Height (m)	1.70 (0.07)	1.84 (0.07)	<0.001	
Weight (kg)	79.0 (14.0)	93.7 (13.3)	<0.001	
Total duration AF (y)	4 [2, 8]	4 [2, 8]	0.681	1.1
Previous catheter ablation n (%)	29 (20.3)	79 (18.5)	0.72	
Myocardial infarction n (%)	2 (1.4)	28 (6.5)	0.03	
AF Type n (%)			0.717	
Paroxysmal AF	52 (36.4)	155 (36.2)		
Persistent AF	91 (63.6)	273 (63.8)		
Serum biomarkers				
CRP (mg/L)	2.00 [0.90, 4.28]	1.30 [0.65, 2.90]	0.006	17.9
Troponin T (ng/L)	6.0 [5.0, 10.0]	9.0 [6.0, 19.75]	<0.001	22.6
NT-proBNP (ng/L)	425 [180, 916]	256 [101, 618]	<0.001	6.8
CHADSVASc				
Congestive HD n (%)	5 (3.5)	34 (7.9)	0.102	
Hypertension n (%)	68 (47.6)	182 (42.5)	0.341	
Age ≥ 65 n (%)	69 (48.3)	127 (29.7)	<0.001	
Age ≥ 75 n (%)	9 (6.3)	8 (1.9)	0.016	
Diabetes mellitus n (%)	6 (4.2)	29 (6.8)	0.362	
Stroke n (%)	17 (11.9)	28 (6.5)	0.061	
Vascular Disease n (%)	10 (7.0)	69 (16.1)	0.009	
Female sex n (%)	143 (100)	0 (0)		
CHA2DSVASc score	2 [1, 3]	1 [0, 2]	<0.001	
Echocardiographic characteristics				
LVEF (%)	53.0 (10.3)	52.0 (10.2)	0.361	14.5
LAVI (mL/m^2^)	42.6 (13.5)	42.0 (12.3)	0.649	9.1
Mitral valve insufficiency n (%)			0.001	1.1
None	13 (9.2)	99 (23.4)		
Mild	85 (59.9)	241 (57.0)		
Moderate	41 (28.9)	77 (18.2)		
Severe	3 (2.1)	6 (1.4)		
Aorta valve insufficiency n (%)			0.52	1.1
None	104 (73.2)	328 (77.5)		
Poor	6 (4.2)	15 (3.5)		
Mild	31 (21.8)	73 (17.3)		
Moderate	1 (0.7)	7 (1.7)		
Antiarrhythmic medication				
Class IA n (%)	8 (5.6)	6 (1.4)	0.013	
Class IC n (%)	41 (28.7)	130 (30.4)	0.78	
Class II n (%)	73 (51.0)	199 (46.5)	0.397	
Class III n (%)	56 (39.2)	191 (44.6)	0.296	
Class IV n (%)	20 (14.0)	54 (12.6)	0.781	
Other medication				
ACE inhibitor n (%)	27 (18.9)	107 (25.0)	0.167	
Loop diuretics n (%)	22 (15.4)	36 (8.4)	0.026	
Thiazide diuretic n (%)	19 (13.3)	47 (11.0)	1	
Angiotensin II antagonist n (%)	30 (21.0)	74 (17.3)	0.387	
Calcium antagonist n (%)	13 (9.1)	42 (9.8)	0.929	
Cholesterol inhibitor n (%)	34 (23.8)	116 (27.1)	0.501	
Nitrates n (%)	3 (2.1)	9 (2.1)	1	
Potassium diuretics n (%)	6 (4.2)	19 (4.4)	1	
OAC n (%)	142 (99.3)	416 (97.2)	0.256	

Values shown as the mean (sd), median [iqr] or n (%). AAD antiarrhythmic drug by Vaughn–Williams class Ia-IV, ACE angiotensin converting enzyme, AF atrial fibrillation, CRP C-reactive protein, HD heart disease, HFpEF = heart failure with preserved ejection fraction, kg kilogram, LAV left atrial volume index, LVEF left ventricular ejection fraction, m meter, mg milligram, ng nano-gram, NT-proBNP n-terminal pro-brain natriuretic peptide, OAC oral anticoagulation, y years.

**Table 2:** Recurrence type.

	Women (n = 143)	Men (n = 428)
Completed 2Y FU	140	403
Freedom of AF	61 (43.6%)	241 (59.8%)
Atrial fibrillation recurrence	26 (18.6%)	76 (18.9%)
Atrial tachycardia/atrial flutter recurrence	53 (37.9 %)	86 (21.3%)

## Data Availability

Data available from the authors upon reasonable request.
